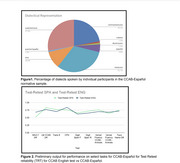# CCAB‐Español: Designing the California Cognitive Assessment Battery in Spanish

**DOI:** 10.1002/alz70857_107603

**Published:** 2025-12-26

**Authors:** Isabella Jaramillo, Kathleen Hall, Lourdes Anllo‐Vento, Kristin Geraci, Michael Blank, Miranda Miranda, Elloise Garcia, Alejandra Ortiz‐Menchaca, Enriqueta Canseco‐Gonzalez, Analia Arevalo, Peter Pebler, David L. Woods

**Affiliations:** ^1^ Neurobehavioral Systems, Inc, Berkeley, CA, USA; ^2^ Universidad de Granada, Granada, Granada, Spain; ^3^ Reed College, Portland, OR, USA; ^4^ University of São Paulo, São Paulo, São Paulo, Brazil

## Abstract

**Background:**

Hispanics are the largest U.S. minority group, expected to grow from 59 to 119 million by 2060 with approximately 25% of Hispanics speaking primarily Spanish. Cognitive test access for U.S. Hispanics remains limited due in part to a lack of Spanish‐speaking examiners, limited access to testing in many rural areas, and the lack of standardized Spanish‐language tests optimized for the US Hispanic population[1].

**Method:**

The CCAB is a standardized, computerized in‐home test with real‐time scoring and data from 1,000+ participants. CCAB‐Español (*n* = 108) mirrors the English version for validity, reliability and comprehensive scoring. Native Spanish‐speaking translators adapted all stimuli, ensuring linguistic and cultural accuracy.

Tests: Test stimuli were adapted to preserve the psychometric characteristics of the English version while ensuring cultural and linguistic relevance for Spanish speakers. For example, the Spanish vocabulary test was adapted by selecting words that matched the English in both difficulty and frequency, with consideration for broad dialectal applicability. ASR: Speech is automatically transcribed using consensus automatic speech recognition (CASR). Linguistic and dialectal variation in Spanish are accommodated for in custom grammars provided by individual ASR engines, and refinements to the Spanish CASR pipeline are continuously made based on normative test data. Scoring design: Scoring files maximize correct response ranges, accounting for dialectal and cultural variations. For example, in the Picture Naming task, “cake” may be identified as *pastel, bizcocho, or torta*, depending on dialect. Gendered and multi‐word responses are similarly accommodated across all scoring files, increasing accuracy and fairness when assessing cognitive function in this population.

**Results:**

The normative sample (*n* = 108; age 18‐79; 62% women) includes 22% Central American, 3% Caribbean, 5% Spanish, 31% Mexican, 28% South American, 10% other Spanish‐speaking adults (bilingual and monolingual) with diverse educational, and socioeconomic backgrounds. All participants completed 27 CCAB‐Español tasks and comprehensive questionnaires. Tests showed strong test/retest reliability (e.g., story recall r = .83), similar to the psychometric properties of the English version.

**Conclusion:**

CCAB‐Español demonstrates high accuracy and reliability for cognitive assessment in Spanish, with psychometric properties similar to the English version. Careful adaptation of stimuli, ASR methods, and scoring accommodates diverse Spanish‐speaking populations.